# Similarities and differences between intermittent and continuous resting-state fMRI

**DOI:** 10.3389/fnhum.2023.1238888

**Published:** 2023-08-03

**Authors:** Justyna Beresniewicz, Frank Riemer, Katarzyna Kazimierczak, Lars Ersland, Alexander R. Craven, Kenneth Hugdahl, Renate Grüner

**Affiliations:** ^1^Department of Biological and Medical Psychology, University of Bergen, Bergen, Norway; ^2^Mohn Medical Imaging and Visualization Centre, Department of Radiology, Haukeland University Hospital, Bergen, Norway; ^3^Department of Clinical Engineering, Haukeland University Hospital, Bergen, Norway; ^4^Division of Psychiatry, Haukeland University Hospital, Bergen, Norway; ^5^Department of Physics and Technology, University of Bergen, Bergen, Norway

**Keywords:** functional Magnetic Resonance Imaging (fMRI), Default Mode Network (DMN), resting state, block design, cognitive tasks

## Abstract

**Introduction:**

Functional Magnetic Resonance Imaging (fMRI) block-design experiments typically include active ON-blocks with presentation of cognitive tasks which are contrasted with OFF- blocks with no tasks presented. OFF-blocks in between ON-blocks can however, also be seen as a proxy for intermittent periods of resting, inducing temporary resting-states. We still do not know if brain activity during such intermittent periods reflects the same kind of resting-state activity as that obtained during a continuous period, as is typically the case in studies of the classic Default Mode Network (DMN). The purpose of the current study was therefore to investigate both similarities and differences in brain activity between intermittent and continuous resting conditions.

**Methods:**

There were 47 healthy participants in the 3T fMRI experiment. Data for the intermittent resting-state condition were acquired from resting-periods in between active task-processing periods in a standard ON-OFF block design, with three different cognitive tasks presented during ON-blocks. Data for the continuous resting-state condition were acquired during a 5 min resting period after the task-design had been presented.

**Results and discussion:**

The results showed that activity was overall similar in the two conditions, but with some differences. These differences were within the DMN network, and for the interaction of DMN with other brain networks. DMN maps showed weak overlap between conditions in the medial prefrontal cortex (MPFC), and in particular for the intermittent compared to the continuous resting-state condition. Moreover, DMN showed strong connectivity with the salience network (SN) in the intermittent resting-state condition, particularly in the anterior insula and the supramarginal gyrus. The observed differences may reflect a “carry-over” effect from task-processing to the next resting-state period, not present in the continuous resting-state condition, causing interference from the ON-blocks. Further research is needed to fully understand the extent of differences between intermittent and continuous resting-state conditions.

## Introduction

Measures of resting-state functional connectivity have been of great interest in the field of neuroimaging for predominantly two reasons: Firstly, studies have provided new insight in understanding the human brain including possible overlaps between functional and structural connectivity (Koch et al., 2002; Greicius et al., 2009). Secondly, studying functional connectivity with resting-state data is relatively straight-forward to apply to studies of brain development and in clinical research. This may lead to improved compliance and lower training demands as compared to other approaches like task-based functional Magnetic Resonance Imaging (fMRI) (Fair et al., 2007). Functional connectivity is typically computed using low-frequency (<0.1 Hz) blood-oxygen-level-dependent (BOLD) signal fluctuations, allowing inference of neuronal activity between regions of interests (ROIs) (Biswal et al., 1995). These low-frequency fluctuations are typically obtained during condition of resting, when the brain consequently being in a resting state. This is thought to represent spontaneous neuronal activation as well as unconstrained mental activity, such as “day-dreaming” or “mind-wandering” (Patriat et al., 2013).

Functional connectivity studies of the resting brain have revealed consistent large-scale spatial distributions of coherent signals which we refer to as Resting State Networks (RSNs). It is now confirmed that these networks reflect functional brain systems supporting core perceptual and cognitive processes (Dijk et al., 2010). Interestingly, although RSNs are mostly mentioned in relation to BOLD fluctuations that emerge spontaneously during resting, the same networks also show synchronous fluctuations in task-paradigms and in altered states of consciousness (Dijk et al., 2010). For example, the default network (DMN), which is the most studied RSN, shows strong correlations during task conditions but at an attenuated level relative to rest (Fransson, 2006). Therefore, these networks may be described as either “task-positive” or “task-negative,” in terms of the direction of correlation between mean network activity and the timing of stimulus-events during a task (Hugdahl et al., 2019). Some studies suggest that performance of a cognitive task might lead to “carry-over effects” onto subsequent resting periods, influencing RSN-connectivity. For example, a study by Waites et al. (2005) on functional connectivity analysis of DMN-regions during resting-periods before and after solving an orthographic lexical retrieval task revealed relatively small increases and decreases in functional connectivity between the resting periods before and after task execution. Pyka et al. (2009) presented evidence that a cognitively challenging working-memory task was followed by increased DMN-activity compared to a non-challenging letter-matching task. This might be interpreted as a functional correlate of self-evaluation and reflection during resting from the preceding task, or as relocation of cognitive resources representing recovery from challenging cognitive demands (Pyka et al., 2009). Additionally, Fox and Raichle (2007) showed that coherent spontaneous brain activity could explain trial−to−trial variance in task paradigms. Hence it is not unreasonable to conclude that intrinsic neuronal activity during resting might have an influence on subsequent task-related behavior and corresponding brain activity. Moreover, Sadaghiani et al. (2010) suggested that ongoing activity is organized in a functional architecture. The evoked neural responses are embedded into an underlying functional architecture which cannot be fully understood in isolation from the context established by ongoing activity.

These observations provide a back-drop for the present study, where the focus was to examine and compare RSN-data acquired during a single prolonged resting period with data acquired during repeated brief resting-periods in between task-processing periods (Riemer et al., 2020). Previous studies have rather investigated how well resting-state activity seen in task-processing experiments reflect resting-state activity, and subsequent RSNs seen in classic resting-state DMN experiments (Fair et al., 2007; Ganger et al., 2015). In our study, we used a standard fMRI block-design, and asked the question of how RSN-activity observed during periods of absences of tasks (OFF-blocks) is different from RSN-activity observed during a prolonged resting-period with no interspersed tasks. Hence, we explored similarities and differences in RSN-activity in two resting-state conditions, an intermittent resting-state condition and a continuous resting-state condition. In particular, we focused on similarities and differences in the Default Mode Network (DMN) between the two experimental conditions since it is one of the most well-known and researched RSNs. We hypothesize that although DMN-activity in principle should be similar between the two conditions, differences in mental states shown in these conditions may influence the recorded DMN-activity and connectivity across the two experimental conditions. In order to compare resting-state data between the two conditions, we first extracted BOLD volumes from the OFF-blocks to create the intermittent resting condition dataset. Secondly, we extracted RSNs from both conditions using Independent Component Analysis (ICA) and identified DMN components that were compared statistically. Thirdly, we compared ROI-to-ROI functional connectivity for the DMN with the regions belonging to selected atlas-networks, using permutation statistics and paired *t*-tests.

## Materials and methods

### Participants

The participants were 47 healthy adults, 27 males and 20 females (mean age 34.9 years, SD 13.9). The participants were recruited in the local Bergen municipality area through general, open announcements. Self-reported absence of any psychiatric and neurological disorder was an inclusion criterion. Before MRI data acquisition, the participants were interviewed for body implants such as pacemakers and any signs of claustrophobia. Furthermore, the participants were presented with an informed consent form to sign. Female participants were additionally asked about pregnancy, which was an exclusion criterion. The study was conducted according to the Declaration of Helsinki regarding ethical standards and in agreement with good clinical practice framework in accordance with all ethical rules and guidelines at our institution for human research and was approved by the Regional Committee for Medical Research Ethics in Western Norway (#2014/1641/REK-Vest). Written informed consent was obtained from all participants.

### Stimuli and design

The task-based fMRI session involved three different cognitive tasks: A mental arithmetic task, a working memory task, and a mental rotation task. These data have been previously reported in a task-related fMRI study (Riemer et al., 2020), therefore parts of the Section “Materials and methods” will be overlapping. There were nine ON-blocks with task presentations (three repetitions of each task), alternated with nine black screen OFF-blocks without task-presentations but with a fixation-cross in the middle of the visual field. The tasks were presented for 34 s in each ON-block with their order changed for each participant and alternated with 34 s OFF-blocks. The timing, duration and sequencing of the stimuli, as well as control of overall timing parameters of the experiment was done in the E-Prime (Psychology Software Tools, Inc., USA^1^) software platform. Presentation of the stimuli with acquisition of the MR data was synchronized using a NordicNeurolab SyncBox (NordicNeuroLab Inc., Norway^2^). The participants were instructed to keep their eyes open during presentation of the OFF-blocks. Each ON- and OFF-block lasted for 68 s, making up a total of 10 min and 2 s. For more detailed description of the stimuli (see Riemer et al., 2020). In the resting-state session participants were asked to lay still and relaxed in the scanner for five min. A fixation-cross was again placed in the middle of the visual field and the participants were instructed to fixate on the cross, “stay awake and do not to think about anything in particular.” For the sake of consistency, the data obtained from the resting-state session of the experiment will be referred to as “continuous resting-state condition data” while the data extracted from the resting (OFF) blocks of the task-based session will be referred to as “intermittent resting-state condition data” in the following. The continuous resting-state data were acquired after the intermittent resting-state data in the experiment.

### MR scanning and data acquisition

Imaging data were collected using a Siemens 3T Magnetom Prisma MR scanner.^3^ An anatomical T1-weighted image was acquired prior to the functional imaging with the following sequence parameters: MPRAGE 3D T1-weighted sagittal volume, TE/TR/TI = 2.28 ms/1.8 s/900 ms, acquisition matrix = 256 × 256 × 192, field of view (FOV) = 256 × 256 mm^2^, 200 Hz/px readout bandwidth, flip angle 8 degrees and total acquisition duration of 7.40 min. All fMRI-data were acquired using 2D gradient echo planar imaging (EPI) with the following parameters: TE/TR = 30 ms/2 s, 306 volumes for the task-based intermittent condition and 150 volumes for the continuous resting state condition, acquisition matrix = 64 × 64, slice thickness = 3.6 mm, 35 slices, FOV = 230 × 230 mm.

### fMRI data processing

Before preprocessing of the task fMRI data, the seven most central 3D volumes were extracted from each of the nine OFF-blocks from the 4D task-fMRI imaging for further processing. Each OFF-block consisted of 17 volumes where the first and last five volumes of each OFF- block were omitted. The first five volumes of the OFF-blocks were omitted to avoid the inclusion of a transient state of the BOLD (Blood Oxygen Level Dependent) signal from ON-blocks to OFF-blocks. We additionally decided to remove the last five OFF-blocks to avoid inclusion of the volumes when participants could anticipate the appearance of the next cognitive task. Pyka et al. (2009) suggest that after cognitively demanding task, such as the n-back task, a non-linear increase in DMN might reflect increased self-referential processes that might be activated by a minor increase in predicting task complexity. Since in our experiment there was a switch between blocks sixteen times after equal time intervals, it is unlikely that a bias would be created by the subject “learning” when the next ON-block was going to appear.

The data extraction procedure was as follows: The intermittent resting-state data were first disentangled into individual volumes using SPM12 (the Welcome Centre for Human Neuroimaging, UCL, London, UK^4^) functions spm vol and spm read vols. Furtherly, the volumes of interest were then saved as separate 3D nifti images using the SPM function spm write vol. The extracted “resting” 3D volumes were finally merged into 4D images consisting of 63 volumes (total time 2 min and 1 s) using SPM function collapse nii scan.^5^ The whole procedure was performed in Matlab R2017b (MathWorks, Natick, MA).

**FIGURE 1:**
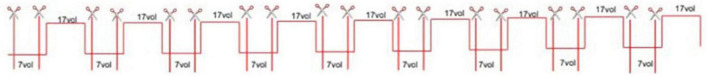
Graphical illustration of the block-design, and the extracted rest-periods during OFF-blocks used for acquiring data for the intermittent resting-condition. The scissors in the figure indicate the seven volumes cut out for analysis of each OFF-block.

The intermittent resting-state condition consisted of nine ON-blocks and nine OFF- blocks, each containing 17 volumes (see Figure 1). The vertical red lines in Figure 1 show where the 4D fMRI image was cut (indicated by scissors above the lines) in order to extract the intermittent resting-state volumes. Seven volumes were cut out from each OFF-block. The first and last five volumes were omitted, seen as being outside of the red vertical lines. Extracted OFF-block volumes were later merged to create 4D fMRI images consisting of 63 OFF-block volumes.

All pre-processing was performed in Matlab R2017b using the CONN functional connectivity toolbox 17b.^6^ Pre-processing consisted of functional realignment and unwarping, functional centering to (0,0,0) coordinates, functional outlier detection (ART-based identification of outlier scans for scrubbing), functional direct segmentation and normalization, structural centering to (0,0,0) coordinates, structural segmentation and normalization and functional smoothing.

### Spatial ICA

Spatial Independent Component Analysis (ICA) was performed separately on resting data from the two conditions. The ICA analysis was performed using Group ICA for fMRI toolbox (GIFT v3.0b). For each condition, 20 independent components were extracted using the algorithm type “Infomax” (Bell and Sejnowski, 1995) with all other settings left as default. Extracted independent components were spatially sorted according to correlation coefficients with the DMN component map template from the 10 components derived in the study by Smith et al. (2009).^7^ This was done to automatically select components which best reflected the DMN in the two data-sets, and to get an overview of the networks present in either condition.

### Conjunction analysis and probability maps

The resultant mean participants’ components with the highest correlation to the template DMN map were used to select individual participants’ components for a second-level conjunction analysis (one-way ANOVA, random effects) across all relative component images for each individual. This allowed quantifying the level of similarity across identified DMN components in the two resting-state conditions. The conjunction analysis was performed in SPM 12. All relevant component maps (one for each participant and condition) were included in the second-order analysis (one-way ANOVA). After estimation of the model, multiple contrasts for comparison were defined. Statistical maps were corrected for multiple comparisons (FWE), and a significance threshold using a *p*-value of 0.05 and cluster extent of 10 voxels were selected for all comparisons. Additionally, to test the spatial overlap of the components with the highest and second-highest correlation to the template DMN map, probability maps for all components and all participants were calculated using SPM 12. Firstly, all components were binarized using a threshold of *z* > 1.96 (equivalent to *p* < 0.05). Secondly, all binarized components were summed up voxel-wise, multiplied by 100 and divided by the total amount of images (images from two components of 47 participants from two conditions). Hence, the displayed voxel values represent the probability (%) of the highest and second-highest DMN-correlated components to be present in all participants in the two conditions.

### Functional connectivity analysis

A region-of-interest (ROI) analysis was performed to explore functional connectivity differences for the DMN and eight selected networks between the two resting-state conditions. The ROI-to-ROI functional connectivity analysis was performed with the CONN toolbox (see text footnote 6). Functional connectivity z- scores were calculated between the four ROIs belonging to DMN: DefaultMode.MPFC (1,55,-3), DefaultMode.LP (L) (-39,-77,33), DefaultMode.LP (R) (47,-67,29), DefaultMode.PCC (1,-61,38), and 32 ROIs belonging to eight selected functional networks (DMN, Sensori/Motor, Visual, Salience, Dorsal Attention, Fronto Parietal, Language, Cerebellar) as defined in the CONN default network atlas. The resulting connectivity matrices for the two conditions were furtherly extracted and used to perform a between-condition comparison using the Permutation Analysis of Linear Models (PALM) in FSL.^8^ The comparison was set up as a two-tailed paired *t*-test and utilized 10,000 permutations. The results were corrected for multiple comparisons using Family Wise Error correction (FWE) (*p* < 0.05).

## Results

### ICA analysis

**FIGURE 2:**
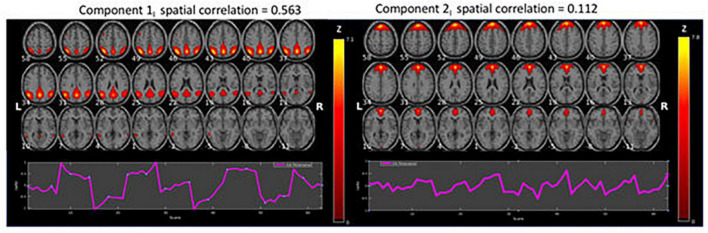
Results of the ICA analysis for intermittent (I) resting-state condition data (*z* > 1.96). The figure shows BOLD activations overlaid on axial anatomy slices from top **(upper left corner)** to bottom **(lower right corner)** in steps of 3 mm, with MNI z-coordinates ranging from 58 to –11. The ROIs related to ICA Component 1 were located bilaterally in the Precuneus/Posterior Cingulate Cortex (PCC), Inferior and Superior Parietal Lobules. The corresponding ROIs related to ICA component 2 were located in the Medial Superior Frontal Gyrus, bilaterally in the Lateral Superior Frontal Gyrus, Anterior Cingulate Cortex (ACC)/Supplementary Motor Area (SMA), and bilaterally in the Middle Temporal Gyrus.

**FIGURE 3:**
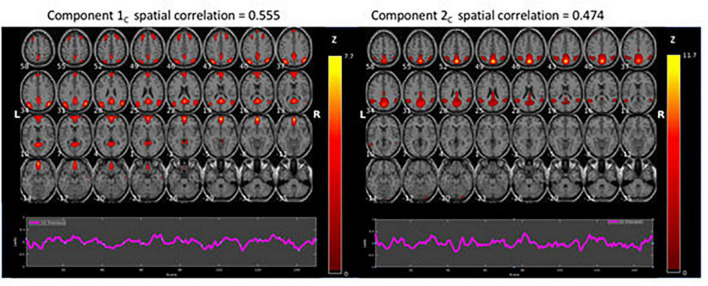
Results of the ICA results for the continuous (C) resting-state condition data (*z* > 1.96). The figure shows BOLD activations overlaid on axial anatomy slices from top **(upper left corner)** to bottom **(lower right corner)** in steps of 3 mm, with MNI z-coordinates ranging from 58 to –35. The ROIs related to ICA component 1 were located bilaterally in Precuneus/Posterior Cingulate Cortex (PCC), the Lateral Superior Frontal Gyrus, and in the Medial Superior Frontal Gyrus/Anterior Cingulate Cortex (ACC). The corresponding ROIs related to ICA Component 2 were located in the Precuneus/Posterior Cingulate Cortex (PCC), bilaterally in the Superior and Inferior Parietal Lobules, and bilaterally in the Middle Temporal Gyrus.

The spatial ICA decomposition of the resting-state data was designed to result in 20 independent component maps for each of the two conditions. After spatial sorting of the components, the two component-maps with the strongest correlation with the template DMN map were selected for each condition, Figures 2, 3.

Figure 2 shows the two components with the highest correlations with the example-DMN map taken from Koch et al. (2002). Below each component is the corresponding time course. The figure shows BOLD activations overlaid on axial anatomy slices from top (upper left corner) to bottom (lower right corner) in steps of 3 mm, with MNI z-coordinates ranging from 58 to −11. The ROIs related to ICA Component 1 were located bilaterally in the Precuneus/Posterior Cingulate Cortex (PCC), Inferior and Superior Parietal Lobules. The corresponding ROIs related to ICA component 2 were located in the Medial Superior Frontal Gyrus, bilaterally in the Lateral Superior Frontal Gyrus, Anterior Cingulate Cortex (ACC)/Supplementary Motor Area (SMA), and bilaterally in the Middle Temporal Gyrus.

Figure 3 shows the two components with the highest correlations with the example-DMN map taken from Koch et al. (2002). Below each component is the corresponding time course. There were significant BOLD activations overlaid on axial anatomy slices from top (upper left corner) to bottom (lower right corner) in steps of 3 mm, with MNI z-coordinates ranging from 58 to −35. The ROIs related to ICA component 1 were located bilaterally in Precuneus/Posterior Cingulate Cortex (PCC), the Lateral Superior Frontal Gyrus, and in the Medial Superior Frontal Gyrus/Anterior Cingulate Cortex (ACC). The corresponding ROIs related to ICA Component 2 were located in the Precuneus/Posterior Cingulate Cortex (PCC), bilaterally in the Superior and Inferior Parietal Lobules, and bilaterally in the Middle Temporal Gyrus.

As seen in Figures 2, 3, although both conditions activated DMN nodes in anterior and posterior brain regions, there was also a slight difference between the two conditions. Figure 2 shows that the ICA-1_I_ component from the intermittent condition primarily activated posterior DMN regions and ICA-2_I_ primarily activated anterior brain regions. The corresponding ICA components in Figure 3 for the continuous condition was both anterior and posterior regions for the ICA-1_C_ component and primarily posterior regions for the ICA-2_C_ component.

### Conjunction analysis

**FIGURE 4:**
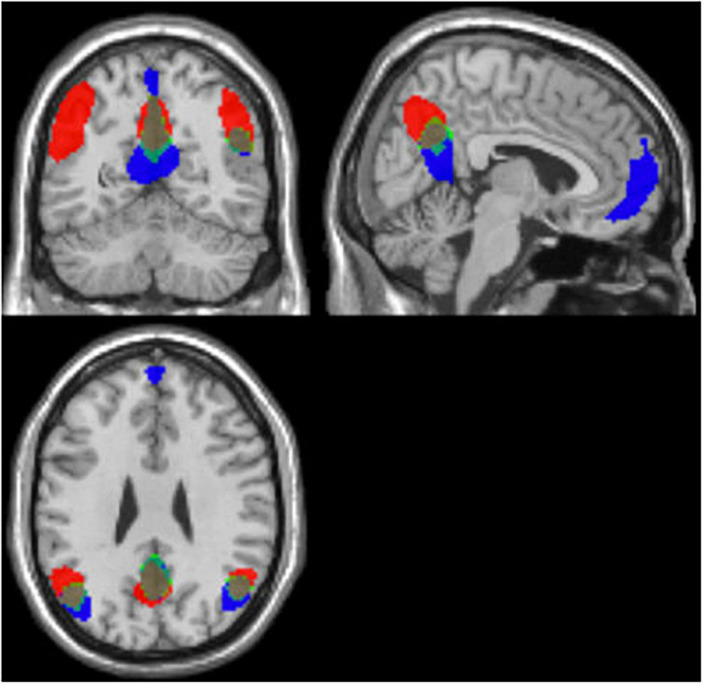
Results of the conjunction (marked in green) analysis for the intermittent (1_I_) marked in red, and continuous (1_C_) marked in blue, components, based on all 47 participants.

**FIGURE 5:**
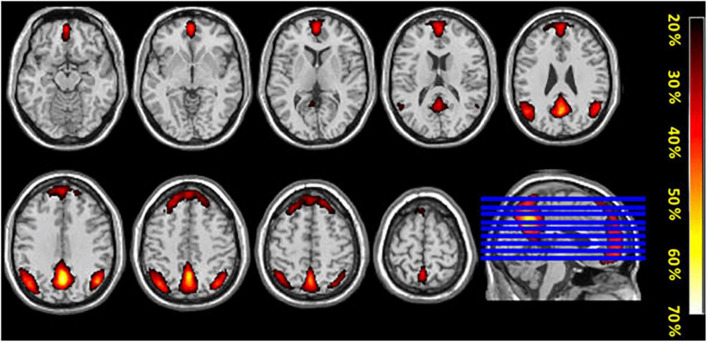
The figure shows mean probability-maps (expressed as%) for all 47 participants for overlap of ICA components obtained from the two conditions (intermittent resting-state and continuous resting-state).

The conjunction analysis using the components with the highest correction with template DMN (1_I_–intermittent resting condition and 1_C_–continuous resting-state condition) for each participant showed substantial spatial overlaps. This indicates that the DMN was activated in both the intermittent and continuous resting-state condition, see Figure 4. Additionally, the probability map was calculated for the components with the highest and second-highest correlation values with the template DMN map, in all 47 participants (1_I_ and 2_I_ components for the intermittent resting-state condition and 1_C_ and 2_C_ for the continuous resting-state condition). The probability map showed that the overlap between components was weaker in the frontal ROI of the DMN while the strongest overlap was in the posterior ROIs, see Figure 5.

### Functional connectivity analysis

**FIGURE 6:**
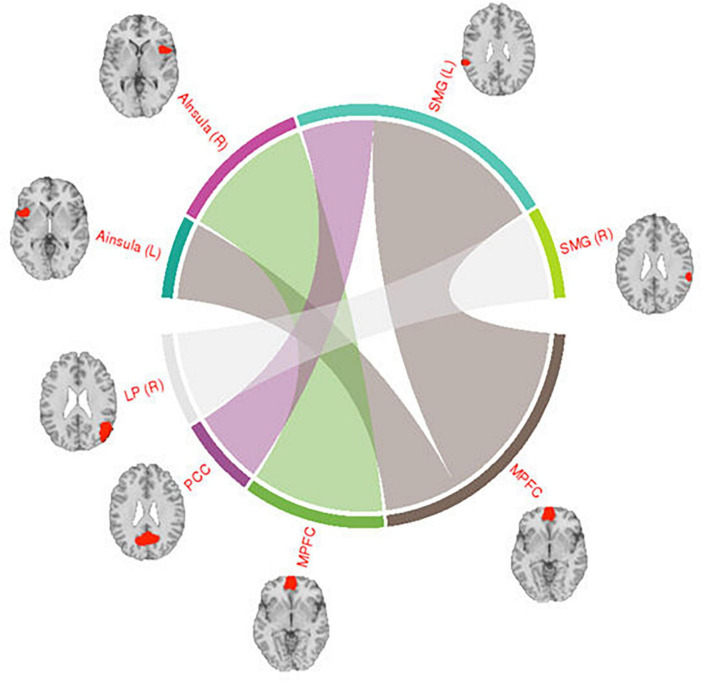
Visualization of connections between the DMN and Salience network seeds. The regions shown in the figure are: Posterior Cingulate Cortex (PCC), Medial Prefrontal Cortex (MPFC), Supramarginal Gyrus (SMG), Anterior Insula (AI), Lateral Parietal (LP), and Posterior Superior Temporal Gyrus (PSTG). The right side of the hemisphere is marked by the letter R and the left side by the letter L.

**FIGURE 7:**
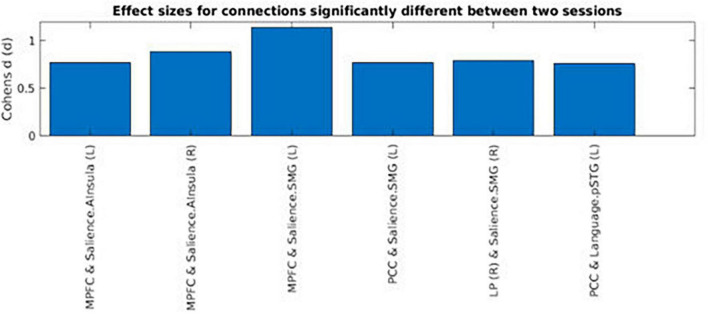
Cohen’s effect-size values for functional connections that were significantly different between the intermittent and the continuous resting-state condition. The regions shown in the figure are: Posterior Cingulate Cortex (PCC), Medial Prefrontal Cortex (MPFC), Supramarginal Gyrus (SMG), Anterior Insula (AI), Lateral Parietal (LP), and Posterior Superior Temporal Gyrus (PSTG). The right side of the hemisphere is marked by the letter R and the left side by the letter L.

Across the two conditions, six connections between DMN network ROIs and ROIs belonging to the other selected networks are shown in Table 1. Interestingly, most of the significantly different connections observed were between seeds of the DMN network and the Salience network (SN) (see Figure 6). To show the direction of the identified differences, Cohn’s *d* effect-size values were calculated (see Figure 7). The presented effect-size values indicate that significant connections between the DMN and the other ROIs were higher for the intermittent than for the continuous resting-state condition. The comparisons were performed as paired two-tailed *t*-tests between conditions. Table 1 shows significantly different connections between the two conditions [*p*^(–log10)^ > 1.301]. Each row shows pairs of ROIs creating a connection.

**TABLE 1:** Comparison of atlas-based DMN ROIs for intermittent and continuous resting-state data.

	ROI pairs		
No	ROI1	ROI2	*p* ^(–log10)^
1	networks.DefaultMode. MPFC (1,55,–3)	networks.Salience. AInsula (L) (–44,13,1)	1.65
2	networks.DefaultMode. MPFC (1,55,–3)	networks.Salience. AInsula (R) (47,14,0)	2.96
3	networks.DefaultMode. MPFC (1,55,–3)	networks.Salience.SMG (L) (–60,–39,31)	4.00
4	networks.DefaultMode. PCC (1,–61,38)	networks.Salience.SMG (L) (–60,–39,31)	1.62
5	networks.DefaultMode. LP (R) (47,67,29)	networks.Salience.SMG (R) (62,–35,32)	1.85
6	networks.DefaultMode. PCC (1,–61,38)	networks.Language. pSTG (L) (–57,–47,15)	1.54

An extended version of the Table, including all comparisons, not only significant ones, can be found in Supplementary material.

The connections shown in Figure 6 were all significantly different between the intermittent and the continuous resting-state condition [*p*^(–log10)^ > 1.3010]. Thickness of the lines in the circular graph in Figure 6 represents degree of the difference for a given connection between the two conditions (*p*-value in logarithmic scale). The anatomical regions presented in the figure are: Posterior Cingulate Cortex (PCC), Medial Prefrontal Cortex (MPFC), Supramarginal Gyrus (SMG), Anterior Insula (AI), Lateral Parietal (LP). The right side of the hemisphere is marked by the letter R and the left side by the letter L.

Figure 7 shows Cohen’s d effect-sizes for significant connections. The results were that for most of the significant differences based on mean functional connectivity between the two conditions, values for the intermittent resting-state condition were higher than the corresponding values for the continuous resting-state condition. The regions presented in Figure 7 are: Posterior Cingulate Cortex (PCC), Medial Prefrontal Cortex (MPFC), Supramarginal Gyrus (SMG), Anterior Insula (AI), Lateral Parietal (LP), and Posterior Superior Temporal Gyrus (PSTG). The right side of the hemisphere is marked by the letter R and the left side by the letter L.

## Discussion

The current study examined similarities and differences in brain networks with data collected using two different experimental conditions, an intermittent resting-state condition and a continuous resting-state condition, with a focus on the Default Mode Network (DMN). Intermittent resting-state data were extracted from rest-periods (OFF-blocks) in between active task-processing periods (ON-blocks). Continuous resting-state condition data were from 5 min with task-absence, i.e., a classic resting-state period. We first used spatial ICA to identify the DMN network, and then examined differences in the overall spatial architecture and connectivity between the ROIs of the DMN and ROIs belonging to other main functional networks of the brain. Our results showed that the DMN network was present in both the intermittent and continuous resting-state condition. Although the overall DMN activity-pattern was similar between the two conditions, there were also some differences. Comparing functional connectivity between ROIs of the DMN and ROIs belonging to other functional networks revealed that the DMN in the intermittent resting-state condition was stronger connected with the SN compared with the corresponding analysis for the DMN observed in the continuous resting-state condition. This is to be expected considering that the DMN in the former condition is switched on and off when switching from active to passive processing (Hugdahl et al., 2019; Riemer et al., 2020).

To our knowledge there are very few studies that have attempted to compare DMN activity from two resting-state conditions. The few studies that did attempt to compare between intermittent and continuous resting-state conditions have mainly focused on the methodological aspect of the comparison (Fair et al., 2007; Ganger et al., 2015). Although this approach is valid for assessing how well different methods perform in extracting “continuous-like” resting-state networks from task-fMRI, it does not address the theoretical issue whether the DMN seen in intermittent versus continuous resting-state reflect two sides of the same network, or two different networks. The current study therefore provides a unique opportunity to compare resting-state networks across two conditions in the same subject acquired in a single MRI session. In fact, a recent study by Zhao et al. (2023) compared fMRI-data from a resting-state condition with data from three different fMRI-tasks. Interestingly, they found that functional connectivity during the task-condition correlated better with behavior than functional connectivity during the resting-condition. The authors concluded that task-based fMRI paradigms may better reflect behavior changes than resting-state fMRI, which we support on the basis of the present data. Although the data collected for the current study were not ideal due to differences in duration between the two conditions, it nevertheless allows for a unique comparison on a within-participants basis, which is a strength of the study. Increasing the duration of the blocks in the block-design to have an equal duration of the two conditions would solve the problem of the duration differences but at the same time might introduce additional challenges. The longer the duration of the acquisition periods, the higher the probability that the participants would learn the tasks. This may in turn impact the level of activation of task-positive networks which are supposedly antagonistic to the DMN, such as the EMN Hugdahl et al. (2015). As was suggested by Hugdahl et al. (2015), the EMN is upregulated during active task-processing, therefore it can be suspected that learning may attenuate this effect. Moreover, one could argue that prolongation of the OFF-blocks would improve data quality in comparison with a continuous resting condition. However, long OFF-blocks are generally not recommended. As Henson (2007) writes: “*For two alternating conditions, for example, block lengths of more than 50 s would cause the majority of signal (i.e., that at the fundamental frequency of the square-wave alternation) to be removed when using a high- pass cut-off of 0.01 Hz. In fact, the optimal block length in an on-off design, regardless of any high-pass filtering, is approximately 16 s”* (Henson, 2007). Thus, although not an ideal design, we nevertheless believe that the current design for comparison between an intermittent and continuous resting situation did produce valid data, inviting to further future research.

The results showed that the overall network connectivity patterns were in line with the results of the few previous studies (Fair et al., 2007; Ganger et al., 2015) despite the different methods used to address the comparison between resting conditions. When comparing ICA components that included DMN activity from both conditions, there was a clear overlap in the overall pattern of activity between the two conditions in both anterior and posterior brain regions. The overlap was present regardless of the method used for the comparison (conjunction or probability maps) and remained the same even after inclusion of ICA-maps characterized by a weaker correlation with the template DMN-map. The highest similarity between the two DMN-maps was observed in the posterior parts of the brain, which also converge with the key DMN-nodes in the precuneus and posterior cingulate cortex, which was expected.

The intermittent resting-state condition resulted in reduced activity in the medial prefrontal cortex (MPFC), and the first intermittent ICA-component (1_I_) did not include the classic DMN frontal node (Shulman et al., 1997). The second ICA-component (2_I_) showed weak correlation with the template DMN, and weakly overlapped with frontal activation components that were obtained in the continuous resting-state condition. This is also seen in the percentage probability maps in Figure 5. When comparing the two ICA-components with strongest correlation to the DMN template in the two conditions (1_C_, 2_C_, 1_I_, and 2_I_), the activity in frontal region was less similar between the intermittent and continuous resting-state conditions. This could mean that the second component (2_I_) obtained from the intermittent resting-state condition is driven by different underlying mechanisms than the corresponding continuous resting-state component.

Activity in the MPFC is involved in self-related processing (referencing information to oneself), retrieving personal knowledge such as autobiographical memories and simulate personal future events or social interactions (Spreng et al., 2009; Andrews-Hanna, 2012; Denny et al., 2012; D’Argembeau, 2013; Moran et al., 2013). This may reflect brain correlates of mind-wandering, which is expected to be dominant in a continuous resting-state condition compared to a intermittent resting-state condition. It may be hypothesized that in an intermittent resting-state state, participants will not achieve a deep state of relaxation and mind wandering, comparable to a continuous resting-state period. Activity during ON-blocks may therefore “carry over” and interfere with activity during OFF-blocks (Riemer et al., 2020). As a result, there would be reduced activity in the MPFC. An interesting question is the degree to which the current results, and particularly the MPFC results, can be generalized to other domains beyond the cognitive domain. The three tasks used in the current study all primarily target cognition, especially attention, working memory and executive control. We are not aware of any studies that have investigated whether similar network relationships would appear for tasks tapping affective domains. It could be speculated that affective tasks might activate basal ganglia nodes to a higher degree than cognitive tasks. What we do know, however, is that an auditory task, dichotic listening to speech sounds, results in correspondingly up- and down-regulation of the DMN and task-positive networks across ON- and OFF-blocks as shown by Hugdahl et al. (2019).

The comparison of DMN functional connectivity between the two resting-state conditions is a test of how network dynamics vary between conditions. There was a significant difference for the correlation between the DMN and the SN networks, with stronger connectivity between the DMN and SN in the intermittent resting-state condition compared to the connectivity in the continuous resting-state condition. This finding supports the hypothesis that the SN is modulating both the central executive network (CEN) and the DMN. The salience network (SN) includes activity in the anterior cingulate cortex (ACC) and the ventral anterior insular cortices, as well as the amygdala, hypothalamus, ventral striatum, thalamus, and specific brainstem nodes. SN is activated in response to diverse cognitive tasks and conditions, suggesting a task non-specific function (Seeley, 2019), also consistent with what Hugdahl et al. (2015), labeled the extrinsic mode network (EMN). The hypothesis that the salience network (SN) plays a significant role in switching between the CEN and DMN has been suggested in previous studies (Mesulam, 1998; Craig, 2002; Critchley et al., 2004; Goulden et al., 2014). The ACC and the insula have known reciprocal connectivity with one another and are also connected to motor and sensory areas of the brain. This makes these regions ideal to receive inputs required to initiate switching between resting and task-processing networks, like switching between the DMN and CEN. These regions are also co-activated in different cognitively demanding tasks (Dosenbach et al., 2006). Therefore, the higher functional connectivity for the DMN with nodes of the SN in the current study may reflect that the SN is dynamically modulating brain activity, while constantly transitioning between active and resting periods during the experiment.

Understanding the role of the SN in modulation of the CEN and DMN networks may be critical for understanding underlying neuronal mechanisms of mental disorders. Dysfunctional “carry-over” effects from one network to another may be characteristic of brain connectivity dynamics in, e.g., schizophrenia (Manoliu et al., 2013). Manoliu et al. (2013) demonstrated increased functional connectivity between the DMN and CEN in schizophrenia patients and related this to severity of hallucinations. Additionally, Hugdahl et al. (2020) suggested that abnormal switching between networks in schizophrenia patients may be the reason for the fluctuating pattern seen in auditory verbal hallucinations, with intermittent periods of presence and absence of “voices.”

Despite all research done on the dynamics of brain activity and DMN activity, we still do not know the functional role of this network and what exact role that different nodes of the DMN play for the dynamics of the network as a whole. Although the DMN was present in both conditions in the current study, there were also differences in overall DMN activity and connectivity between the two conditions. This has also been pointed out in previous studies, e.g., Pyka et al. (2009), who used temporal ICA and showed that the DMN was not only more down-regulated during performance of more demanding tasks (working memory 2-back compared to 1-back task), but that it was also more activated after performance of such tasks. This led to the hypothesis that cognitive load influences DMN-activity during task performance, and in particular after performance of challenging or demanding tasks (Pyka et al., 2009). The fact that similar “carry-over” effects were seen in the present study may indicate that the DMN during brief intermittent resting-periods may be more easily disrupted than the “classic” DMN seen in prolonged resting-periods without any intermittent task-processing periods. At the same time, “classic” resting-state designs with 10–20 min of uninterrupted resting while in the scanner, may not reflect a typical situation in daily life since such long resting periods are unusual in modern urban life. Experimental designs that are distant from everyday life dynamics may be a hindrance to progress of understanding the neurobiology of the brain and brain-related mental disorders.

### Limitations

A Previous study by Fair et al. (2007) used different strategies for volume extraction than the current study: “*At the start of a BOLD run steady state was assumed after 4 frames (∼10 s) and for each run these frames were removed on a voxel-wise basis. Two frames after the start of each task block (∼5 s) were included as resting state to account for the hemodynamic delay. At the end of each task block, 6 frames (∼15 s) were excluded from the resting state data, allowing for the hemodynamic response to return to baseline” (Fair et al., 2007).* We decided to not replicate this methodology in our study for several reasons. Firstly, including the volumes from the end of the OFF-blocks or beginning of the ON-blocks does impose the risk of confounding the intermittent resting state condition data due to participants “anticipation” for the ON-block to start and the cognitive “preparations” processes that might emerge during that time of the experiment. Following the strategy presented by Fair et al. (2007) we could increase the length of the intermittent resting state condition only if we include the two first volumes of the following ON-blocks, otherwise accounting for the additional difference in TR parameters between two studies, the number of volumes between studies would be similar.

The OFF-block in the intermittent resting-state condition were extracted out of 17 whole brain volumes (34 s). This limits the possible number of volumes to create 4D intermittent resting-state condition data. Thus, the intermittent resting-state condition data were of relatively shorter duration than the continuous resting-state data. However, continuous resting-state data were in themselves of relatively short duration (5 min). A problem that emerges from block-fMRI experiments with relatively short resting OFF-blocks is that it limits the range of available frequencies to extract resting-state information (Fair et al., 2007). However, a study by Salvador et al. (2005) showed that even when isolating higher frequencies (>0.1 Hz) this produced similar correlation profiles, but with slightly lower correlations than when using frequencies lower than 0.1 Hz. Therefore, concatenating resting-state epochs in a block-design may provide similar correlation profiles as profiles obtained with continuous resting-state condition data, even though the lowest frequency components may be missing due to a shorter total sampling period. The method used to create 4D intermittent resting-state data also induced an artifact in the points in which data from the OFF-blocks were merged together. However, these artifacts were present periodically and should not significantly have impacted our results. Related to this is the possibility that the strength of connectivity differed between condition as a consequence of the length of the time-series. As a check we computed effect-sizes for comparisons between the two conditions for all nodes, not only the significant ones. The results showed that there is a trend across a number of nodes. However, it is also clear that this is not common to all nodes, and that the degree and direction of the relation appears to cluster according to discrete functional networks, see Supplementary Figure 1. It is therefore highly unlikely that the reduced number of timepoints in the intermittent resting-state would give rise to significantly *stronger* connectivity on account of data quality, or that trends in connectivity would cluster according to discrete functional networks as observed here.

## Conclusion and future directions

The DMN network observed during OFF-blocks in the intermittent resting-state condition seems to share a similar pattern with the DMN network obtained during a continuous resting-state condition. However, there were also some differences between the conditions, mainly in frontal areas of the network. The functional connectivity analysis between DMN nodes and the whole brain volume revealed further differences between the two conditions. From inspection of Figure 7, it seems that the DMN in the intermittent resting-state condition had significantly stronger connections with parts of the SN, being previously associated with modulating switching between the DMN and CEN. The observed differences may reflect a “carry over”-effect from task-processing ON-blocks to a following OFF-blocks. However, further research is needed to fully understand the extend of the differences between these two conditions, and whether the DMN is essentially similar in an intermittent and a continuous resting-state situation. It could be of interest to examine the dynamics of changes in the DMN network over longer periods of activity and resting. It could also be of interest to investigate if the “carry-over”- effect is task-dependent, by varying task difficulty and cognitive load.

## Data availability statement

The data analyzed in this study is subject to the following licenses/restrictions: Due to restrictions posed by the ethics approval, data are not allowed shared other than after request to the corresponding author, and after written permission from the ethics committee. Requests to access these datasets should be directed to KH, hugdahl@uib.no.

## Ethics statement

The studies involving human participants were reviewed and approved by the Regional Committee for Medical Research Ethics in Western Norway (2014/1641/REK Vest). Written informed consent was obtained from all participants. The patients/participants provided their written informed consent to participate in this study.

## Author contributions

JB development of the fMRI tasks, data collection, analysis of the data, manuscript writing, and figure preparation. FR assisted in data analysis. KK development of fMRI tasks and data collection. LE and KH conceived and conducted the experiments. AC contributed to analysis of the data and read and commented on the manuscript. RG planned and conceived the study and conducted the experiment. All authors reviewed the manuscript.

## Footnotes

1
https://pstnet.com/

2
https://nordicneurolab.com/

3
https://www.siemens-healthineers.com/magnetic-resonance-imaging

4
https://www.fil.ion.ucl.ac.uk/spm/

5
https://in.mathworks.com/matlabcentral/fileexchange/8797-tools-for-nifti-and-analyze-image

6
https://web.conn-toolbox.org

7
https://www.fmrib.ox.ac.uk/datasets/brainmap+rsns/

8
https://fsl.fmrib.ox.ac.uk/fsl/fslwiki/PALM
