# Supplementary material for: Similarities and differences between intermittent and continuous resting-state fMRI
Source: Front Hum Neurosci. 2023 Aug 3;17:1238888. doi: 10.3389/fnhum.2023.1238888 (PMC10435290; doi:10.3389/fnhum.2023.1238888)
Supplement: Supplementary file 1 [file Data_Sheet_1.PDF]

Supplementary materials

Extended Table 1 including all comparisons (significant comparisons are highlighted in bold)

No	ROI1	ROI2	-log10(p)
1	DefaultMode.MPFC (1,55,-3)	SensoriMotor.Lateral (L) (-55,-12,29)	0,2525
2	DefaultMode.LP (L) (-39,-77,33)	SensoriMotor.Lateral (L) (-55,-12,29)	0
3	DefaultMode.LP (R) (47,-67,29)	SensoriMotor.Lateral (L) (-55,-12,29)	0
4	DefaultMode.PCC (1,-61,38)	SensoriMotor.Lateral (L) (-55,-12,29)	0
5	DefaultMode.MPFC (1,55,-3)	SensoriMotor.Lateral (R) (56,-10,29)	0,5764
6	DefaultMode.LP (L) (-39,-77,33)	SensoriMotor.Lateral (R) (56,-10,29)	0,0626
7	DefaultMode.LP (R) (47,-67,29)	SensoriMotor.Lateral (R) (56,-10,29)	0,6609
8	DefaultMode.PCC (1,-61,38)	SensoriMotor.Lateral (R) (56,-10,29)	0,0134
9	DefaultMode.MPFC (1,55,-3)	SensoriMotor.Superior (0,-31,67)	0
10	DefaultMode.LP (L) (-39,-77,33)	SensoriMotor.Superior (0,-31,67)	0
11	DefaultMode.LP (R) (47,-67,29)	SensoriMotor.Superior (0,-31,67)	0
12	DefaultMode.PCC (1,-61,38)	SensoriMotor.Superior (0,-31,67)	0
13	DefaultMode.MPFC (1,55,-3)	Visual.Lateral (L) (-37,-79,10)	0
14	DefaultMode.LP (L) (-39,-77,33)	Visual.Lateral (L) (-37,-79,10)	0
15	DefaultMode.LP (R) (47,-67,29)	Visual.Lateral (L) (-37,-79,10)	0
16	DefaultMode.PCC (1,-61,38)	Visual.Lateral (L) (-37,-79,10)	0,0005
17	DefaultMode.MPFC (1,55,-3)	Visual.Lateral (R) (38,-72,13)	0
18	DefaultMode.LP (L) (-39,-77,33)	Visual.Lateral (R) (38,-72,13)	0
19	DefaultMode.LP (R) (47,-67,29)	Visual.Lateral (R) (38,-72,13)	0
20	DefaultMode.PCC (1,-61,38)	Visual.Lateral (R) (38,-72,13)	0
21	DefaultMode.MPFC (1,55,-3)	Salience.ACC (0,22,35)	0
22	DefaultMode.LP (L) (-39,-77,33)	Salience.ACC (0,22,35)	0,0026
23	DefaultMode.LP (R) (47,-67,29)	Salience.ACC (0,22,35)	0,0169
24	DefaultMode.PCC (1,-61,38)	Salience.ACC (0,22,35)	0
**25**	**DefaultMode.MPFC (1,55,-3)**	**Salience.AInsula (L) (-44,13,1)**	**1,6536**
26	DefaultMode.LP (L) (-39,-77,33)	Salience.AInsula (L) (-44,13,1)	0,3103
27	DefaultMode.LP (R) (47,-67,29)	Salience.AInsula (L) (-44,13,1)	0,1494
28	DefaultMode.PCC (1,-61,38)	Salience.AInsula (L) (-44,13,1)	0,2494
**29**	**DefaultMode.MPFC (1,55,-3)**	**Salience.AInsula (R) (47,14,0)**	**2,9586**
30	DefaultMode.LP (L) (-39,-77,33)	Salience.AInsula (R) (47,14,0)	0,0157
31	DefaultMode.LP (R) (47,-67,29)	Salience.AInsula (R) (47,14,0)	0,1608
32	DefaultMode.PCC (1,-61,38)	Salience.AInsula (R) (47,14,0)	0,8804
33	DefaultMode.MPFC (1,55,-3)	Salience.RPFC (L) (-32,45,27)	0
34	DefaultMode.LP (L) (-39,-77,33)	Salience.RPFC (L) (-32,45,27)	0
35	DefaultMode.LP (R) (47,-67,29)	Salience.RPFC (L) (-32,45,27)	0
36	DefaultMode.PCC (1,-61,38)	Salience.RPFC (L) (-32,45,27)	0
37	DefaultMode.MPFC (1,55,-3)	Salience.RPFC (R) (32,46,27)	0
38	DefaultMode.LP (L) (-39,-77,33)	Salience.RPFC (R) (32,46,27)	0
39	DefaultMode.LP (R) (47,-67,29)	Salience.RPFC (R) (32,46,27)	0
40	DefaultMode.PCC (1,-61,38)	Salience.RPFC (R) (32,46,27)	0
**41**	**DefaultMode.MPFC (1,55,-3)**	**Salience.SMG (L) (-60,-39,31)**	**4**
42	DefaultMode.LP (L) (-39,-77,33)	Salience.SMG (L) (-60,-39,31)	0,0002
43	DefaultMode.LP (R) (47,-67,29)	Salience.SMG (L) (-60,-39,31)	0,0095
**44**	**DefaultMode.PCC (1,-61,38)**	**Salience.SMG (L) (-60,-39,31)**	**1,618**
45	DefaultMode.MPFC (1,55,-3)	Salience.SMG (R) (62,-35,32)	0,8945
46	DefaultMode.LP (L) (-39,-77,33)	Salience.SMG (R) (62,-35,32)	0,2104
**47**	**DefaultMode.LP (R) (47,-67,29)**	**Salience.SMG (R) (62,-35,32)**	**1,8539**
48	DefaultMode.PCC (1,-61,38)	Salience.SMG (R) (62,-35,32)	1,1403
49	DefaultMode.MPFC (1,55,-3)	DorsalAttention.FEF (L) (-27,-9,64)	0
50	DefaultMode.LP (L) (-39,-77,33)	DorsalAttention.FEF (L) (-27,-9,64)	0
51	DefaultMode.LP (R) (47,-67,29)	DorsalAttention.FEF (L) (-27,-9,64)	0
52	DefaultMode.PCC (1,-61,38)	DorsalAttention.FEF (L) (-27,-9,64)	0
53	DefaultMode.MPFC (1,55,-3)	DorsalAttention.FEF (R) (30,-6,64)	0
54	DefaultMode.LP (L) (-39,-77,33)	DorsalAttention.FEF (R) (30,-6,64)	0
55	DefaultMode.LP (R) (47,-67,29)	DorsalAttention.FEF (R) (30,-6,64)	0
56	DefaultMode.PCC (1,-61,38)	DorsalAttention.FEF (R) (30,-6,64)	0
57	DefaultMode.MPFC (1,55,-3)	DorsalAttention.IPS (L) (-39,-43,52)	0
58	DefaultMode.LP (L) (-39,-77,33)	DorsalAttention.IPS (L) (-39,-43,52)	0
59	DefaultMode.LP (R) (47,-67,29)	DorsalAttention.IPS (L) (-39,-43,52)	0
60	DefaultMode.PCC (1,-61,38)	DorsalAttention.IPS (L) (-39,-43,52)	0,0004
61	DefaultMode.MPFC (1,55,-3)	DorsalAttention.IPS (R) (39,-42,54)	0,0605
62	DefaultMode.LP (L) (-39,-77,33)	DorsalAttention.IPS (R) (39,-42,54)	0
63	DefaultMode.LP (R) (47,-67,29)	DorsalAttention.IPS (R) (39,-42,54)	0
64	DefaultMode.PCC (1,-61,38)	DorsalAttention.IPS (R) (39,-42,54)	0
65	DefaultMode.MPFC (1,55,-3)	FrontoParietal.LPFC (L) (-43,33,28)	0
66	DefaultMode.LP (L) (-39,-77,33)	FrontoParietal.LPFC (L) (-43,33,28)	0
67	DefaultMode.LP (R) (47,-67,29)	FrontoParietal.LPFC (L) (-43,33,28)	0
68	DefaultMode.PCC (1,-61,38)	FrontoParietal.LPFC (L) (-43,33,28)	0
69	DefaultMode.MPFC (1,55,-3)	FrontoParietal.PPC (L) (-46,-58,49)	0
70	DefaultMode.LP (L) (-39,-77,33)	FrontoParietal.PPC (L) (-46,-58,49)	0,0075
71	DefaultMode.LP (R) (47,-67,29)	FrontoParietal.PPC (L) (-46,-58,49)	0,8729
72	DefaultMode.PCC (1,-61,38)	FrontoParietal.PPC (L) (-46,-58,49)	0,3393
73	DefaultMode.MPFC (1,55,-3)	FrontoParietal.LPFC (R) (41,38,30)	0
74	DefaultMode.LP (L) (-39,-77,33)	FrontoParietal.LPFC (R) (41,38,30)	0
75	DefaultMode.LP (R) (47,-67,29)	FrontoParietal.LPFC (R) (41,38,30)	0
76	DefaultMode.PCC (1,-61,38)	FrontoParietal.LPFC (R) (41,38,30)	0
77	DefaultMode.MPFC (1,55,-3)	FrontoParietal.PPC (R) (52,-52,45)	0
78	DefaultMode.LP (L) (-39,-77,33)	FrontoParietal.PPC (R) (52,-52,45)	0,0424
79	DefaultMode.LP (R) (47,-67,29)	FrontoParietal.PPC (R) (52,-52,45)	0,5461
80	DefaultMode.PCC (1,-61,38)	FrontoParietal.PPC (R) (52,-52,45)	0,0563
81	DefaultMode.MPFC (1,55,-3)	Language.IFG (L) (-51,26,2)	0,117
82	DefaultMode.LP (L) (-39,-77,33)	Language.IFG (L) (-51,26,2)	0,0139
83	DefaultMode.LP (R) (47,-67,29)	Language.IFG (L) (-51,26,2)	0,0112
84	DefaultMode.PCC (1,-61,38)	Language.IFG (L) (-51,26,2)	0,5274
85	DefaultMode.MPFC (1,55,-3)	Language.IFG (R) (54,28,1)	0,3606
86	DefaultMode.LP (L) (-39,-77,33)	Language.IFG (R) (54,28,1)	0,0003
87	DefaultMode.LP (R) (47,-67,29)	Language.IFG (R) (54,28,1)	0
88	DefaultMode.PCC (1,-61,38)	Language.IFG (R) (54,28,1)	0,0188
89	DefaultMode.MPFC (1,55,-3)	Language.pSTG (L) (-57,-47,15)	0
90	DefaultMode.LP (L) (-39,-77,33)	Language.pSTG (L) (-57,-47,15)	0,9076
91	DefaultMode.LP (R) (47,-67,29)	Language.pSTG (L) (-57,-47,15)	0,9555
**92**	**DefaultMode.PCC (1,-61,38)**	**Language.pSTG (L) (-57,-47,15)**	**1,5391**
93	DefaultMode.MPFC (1,55,-3)	Language.pSTG (R) (59,-42,13)	0,0131
94	DefaultMode.LP (L) (-39,-77,33)	Language.pSTG (R) (59,-42,13)	0,3831

No	ROI1	ROI2	-log10(p)
95	DefaultMode.LP (R) (47,-67,29)	Language.pSTG (R) (59,-42,13)	0,2577
96	DefaultMode.PCC (1,-61,38)	Language.pSTG (R) (59,-42,13)	0
97	DefaultMode.MPFC (1,55,-3)	Cerebellar.Anterior (0,-63,-30)	0
98	DefaultMode.LP (L) (-39,-77,33)	Cerebellar.Anterior (0,-63,-30)	0
99	DefaultMode.LP (R) (47,-67,29)	Cerebellar.Anterior (0,-63,-30)	0,2423
100	DefaultMode.PCC (1,-61,38)	Cerebellar.Anterior (0,-63,-30)	0,2566
101	DefaultMode.MPFC (1,55,-3)	Cerebellar.Posterior (0,-79,-32)	0
102	DefaultMode.LP (L) (-39,-77,33)	Cerebellar.Posterior (0,-79,-32)	0
103	DefaultMode.LP (R) (47,-67,29)	Cerebellar.Posterior (0,-79,-32)	0
104	DefaultMode.PCC (1,-61,38)	Cerebellar.Posterior (0,-79,-32)	0
105	DefaultMode.MPFC (1,55,-3)	DefaultMode.MPFC (1,55,-3)	0
106	DefaultMode.LP (L) (-39,-77,33)	DefaultMode.MPFC (1,55,-3)	0
107	DefaultMode.LP (R) (47,-67,29)	DefaultMode.MPFC (1,55,-3)	0
108	DefaultMode.PCC (1,-61,38)	DefaultMode.MPFC (1,55,-3)	0
109	DefaultMode.MPFC (1,55,-3)	DefaultMode.LP (L) (-39,-77,33)	0
110	DefaultMode.LP (L) (-39,-77,33)	DefaultMode.LP (L) (-39,-77,33)	0
111	DefaultMode.LP (R) (47,-67,29)	DefaultMode.LP (L) (-39,-77,33)	0
112	DefaultMode.PCC (1,-61,38)	DefaultMode.LP (L) (-39,-77,33)	0
113	DefaultMode.MPFC (1,55,-3)	DefaultMode.LP (R) (47,-67,29)	0
114	DefaultMode.LP (L) (-39,-77,33)	DefaultMode.LP (R) (47,-67,29)	0
115	DefaultMode.LP (R) (47,-67,29)	DefaultMode.LP (R) (47,-67,29)	0
116	DefaultMode.PCC (1,-61,38)	DefaultMode.LP (R) (47,-67,29)	0
117	DefaultMode.MPFC (1,55,-3)	DefaultMode.PCC (1,-61,38)	0
118	DefaultMode.LP (L) (-39,-77,33)	DefaultMode.PCC (1,-61,38)	0
119	DefaultMode.LP (R) (47,-67,29)	DefaultMode.PCC (1,-61,38)	0
120	DefaultMode.PCC (1,-61,38)	DefaultMode.PCC (1,-61,38)	0
121	DefaultMode.MPFC (1,55,-3)	Visual.Medial (2,-79,12)	0
122	DefaultMode.LP (L) (-39,-77,33)	Visual.Medial (2,-79,12)	0
123	DefaultMode.LP (R) (47,-67,29)	Visual.Medial (2,-79,12)	0
124	DefaultMode.PCC (1,-61,38)	Visual.Medial (2,-79,12)	0
125	DefaultMode.MPFC (1,55,-3)	Visual.Occipital (0,-93,-4)	0,0022
126	DefaultMode.LP (L) (-39,-77,33)	Visual.Occipital (0,-93,-4)	0
127	DefaultMode.LP (R) (47,-67,29)	Visual.Occipital (0,-93,-4)	0,0002
128	DefaultMode.PCC (1,-61,38)	Visual.Occipital (0,-93,-4)	0
