# Supplementary figures and images for: Similarities and differences between intermittent and continuous resting-state fMRI
Source: Front Hum Neurosci. 2023 Aug 3;17:1238888. doi: 10.3389/fnhum.2023.1238888 (PMC10435290; doi:10.3389/fnhum.2023.1238888)

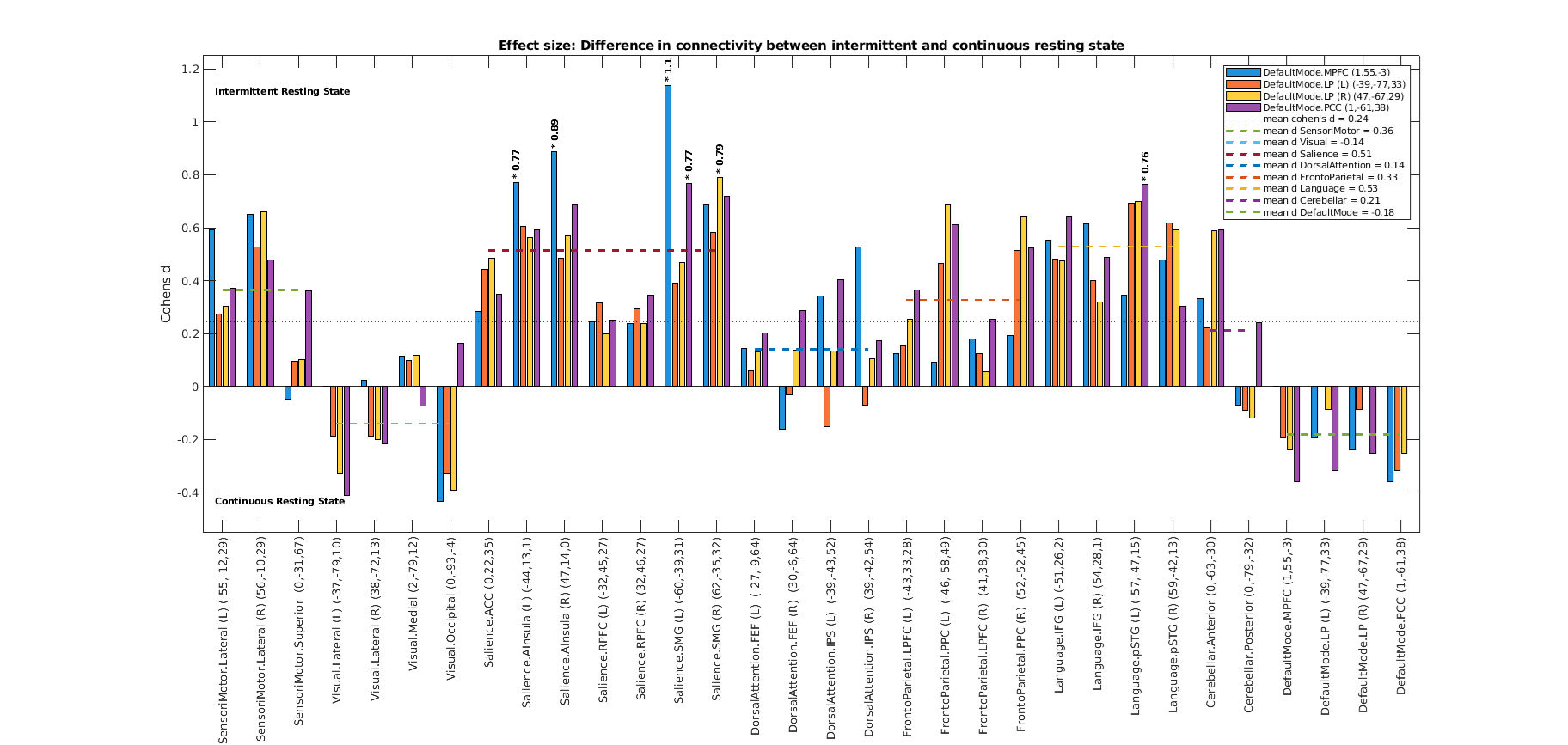

Supplement: Supplementary file 2 [file Image_1.JPEG]
